# Scanning SNPs from a large set of expressed genes to assess the impact of artificial selection on the undomesticated genetic diversity of white spruce

**DOI:** 10.1111/j.1752-4571.2012.00242.x

**Published:** 2012-02-07

**Authors:** Marie-Claire Namroud, Jean Bousquet, Trevor Doerksen, Jean Beaulieu

**Affiliations:** 1 Arborea and Canada Research Chair in Forest and Environmental Genomics, Centre for Forest Research and Institute for Systems and Integrative Biology, Université LavalQuebec City, QC, Canada; 2 Natural Resources Canada, Canadian Forest Service, Canadian Wood Fibre CentreQuebec City, QC, Canada

**Keywords:** *Picea glauca*, conifer and tree breeding, selection intensity, sample size, association genomics, linkage disequilibrium

## Abstract

A scan involving 1134 single-nucleotide polymorphisms (SNPs) from 709 expressed genes was used to assess the potential impact of artificial selection for height growth on the genetic diversity of white spruce. Two case populations of different sizes simulating different family selection intensities (*K* = 13% and 5%, respectively) were delineated from the Quebec breeding program. Their genetic diversity and allele frequencies were compared with those of control populations of the same size and geographic origin to assess the effect of increasing the selection intensity. The two control populations were also compared to assess the effect of reducing the sampling size. On one hand, in all pairwise comparisons, genetic diversity parameters were comparable and no alleles were lost in the case populations compared with the control ones, except for few rare alleles in the large case population. Also, the distribution of allele frequencies did not change significantly (*P* ≤ 0.05) between the populations compared, but ten and nine SNPs (0.8%) exhibited significant differences in frequency (*P* ≤ 0.01) between case and control populations of large and small sizes, respectively. Results of association tests between breeding values for height at 15 years of age and these SNPs supported the hypothesis of a potential effect of selection on the genes harboring these SNPs. On the other hand, contrary to expectations, there was no evidence that selection induced an increase in linkage disequilibrium in genes potentially affected by selection. These results indicate that neither the reduction in the sampling size nor the increase in selection intensity was sufficient to induce a significant change in the genetic diversity of the selected populations. Apparently, no loci were under strong selection pressure, confirming that the genetic control of height growth in white spruce involves many genes with small effects. Hence, selection for height growth at the present intensities did not appear to compromise background genetic diversity but, as predicted by theory, effects were detected at a few gene SNPs harboring intermediate allele frequencies.

## Introduction

Commercial plantations have been established in numerous countries to respond to the increasing demand for forest products (Carle and Holmgren 2008). Reforestation programs for economically important species are generally conducted using planting stock developed through breeding programs. Under certain circumstances, tree breeders are concerned with the necessity to maintain genetic diversity to control inbreeding build-up in future generations and to cope with major environmental disturbances such as climate change (Eriksson et al. 1973; Charlesworth and Willis 2009). However, they are usually focusing on common alleles, as intermediate-frequency alleles provide most of the gain in early rounds of selection (Namkoong et al. 1988; Yanchuk 2001).

When assembling their breeding populations and making selections for next generations, tree breeders must determine the optimum size of these populations and estimate the potential effect of their decisions on the level of genetic diversity maintained. Based on population genetics theory, this question can be addressed from two different perspectives: (i) the reduction in genetic diversity from sampling effects, which should affect all genes more or less equally, and (ii) the reduction in genetic diversity from selection intensity, which should affect only genes implicated in the selected phenotypic trait (Hartl and Clark 1997). By drawing on a small number of individuals, the breeder is faced with the risk of losing some of the alleles or reducing genetic diversity, which might impact the ability to respond to selection pressures for the traits of interest over the next generations. As a result, it appears important to estimate the impact of sampling intensity on allele frequencies because of potential short-term and long-term undesirable lasting effects. The second perspective from which this question can be analyzed is that of selection intensity. As directional selection is expected to drive gene frequencies to an extreme in any finite population (Namkoong et al. 2000), it can be anticipated that by increasing the intensity of selection (i.e., retaining a number of trees with a higher average trait value), gene frequencies at loci under artificial selection will change and some alleles might be more or less rapidly driven toward fixation, depleting genetic variance for the polymorphic loci affecting the economic trait of interest.

Studies were conducted in the past for a number of forest tree species with the aim to compare genetic diversity between natural and breeding populations (Adams 1983; Szmidt and Muona 1985; Knowles 1985; Cheliak et al. 1988; Muona and Hariu 1989; Bergmann and Ruetz 1991; Desponts et al. 1993; Chaisurisri and El-Kassaby 1994; El-Kassaby and Ritland 1996). Globally, these studies did not reveal any significant differences between these types of populations, whether the man-made populations were the result of phenotypic or genetic selection. Some common features of these studies are that they were based on a handful of allozyme markers, comparing populations of different sampling sizes and providing no clear information about the selection intensity applied. This can raise the question about the potential conflicting or overlapping roles of sampling sizes and selection intensities in determining the results of these studies. Given the very small loci sampling in these studies (a few dozens), it also raises the question as to whether the absence of any significant differences between natural and selected populations is related to the fact that the markers used were simply neutral or nearly neutral (e.g. Jaramillo-Correa et al. 2001), bearing no relationship with genes of functional importance whose frequencies are potentially affected by selection. This is especially important because quantitative characters such as height growth, which is one of the main traits for which selection is made by tree breeders, have been shown to be controlled by many genes dispersed throughout the genome each with mainly small effects (Grattapaglia and Kirst 2008; Rae et al. 2008; Freeman et al. 2009; Grattapaglia et al. 2009; Pelgas et al. 2011). This trend appears to hold for a pleiade of other characters related to wood in white spruce (Beaulieu et al. 2011). Also, tree growth traits are generally correlated with each other, and it has been shown that pleiotropic effects are present, with co-locating genomic regions for different characters (e.g., Dillen et al. 2009; Pelgas et al. 2011). These trends highlight the need for a more systematic sampling design and wider genome coverage to enable the detection of allelic variations potentially related to sampling effects or selection intensity.

White spruce [*Picea glauca* (Moench) Voss.] is a boreal conifer species with transcontinental range in North America from Newfoundland to British Columbia, and it extends to the Lake States and New England in the United States (Nienstaedt and Zasada 1990). Because of its fiber attributes, it is considered one of the most important species for lumber and paper industries in Canada (Farrar 1995). Investigations regarding the genetic diversity of the species were initiated in the early 1950s in various regions of Canada, including a dozen provenance tests that were set up in the late 1950s and early 1960s in Quebec (Beaulieu 1994). Based on early results revealing significant variation in economic traits at the geographic (Corriveau and Boudoux 1971) and family levels (Dhir 1976), several breeding programs in different jurisdictions were initiated. Additional provenance/progeny tests were established in the following decades (Beaulieu 1996) and one or two breeding cycles have since been completed.

In the present study, we aimed to test whether genome-wide sampling effects and gene-specific impacts of artificial selection on gene frequencies can be disentangled at an early stage of domestication using samples collected in a first-generation white spruce breeding population and in natural populations from which the trees of the breeding population originated. To do so, we used a genome scan based on single-nucleotide polymorphisms (SNP) located in a large number of expressed genes distributed across the 12 linkage groups of the spruce genome (Pavy et al. 2008). On the one hand, the scanning of hundreds of genes and SNPs should increase the chances of detecting genes involved in growth and potentially affected by selection (Namroud et al. 2008). On the other hand, scanning multiple genes from different ontology classes and with different functional properties minimizes the bias that may result from analyzing genes involved in only one type of function when assessing the impact of selection on genetic diversity (as for most previous enzyme-based diversity studies).

## Materials and methods

### Assembly of case and control populations

The complete details about the breeding strategy applied to develop genetically improved stock for white spruce [*Picea glauca* (Moench) Voss.] in Quebec can be found in the study of Beaulieu (1996). Briefly, the strategy consisted in selecting trees with the best characteristics for lumber and pulp industries from multiple natural populations to form the first-generation breeding population. Three series of genecological tests were first established in Quebec in the 1970s and 1980s. They included 550 open-pollinated families from 120 different populations (provenances). For neutral gene markers, these white spruce populations show non-significant genetic structure and geographic differentiation (e.g., Jaramillo-Correa et al. 2001; Namroud et al. 2008; Beaulieu et al. 2011). When the trees were about 15 years old, the families with the highest breeding values in each of the series were selected. The breeding values were derived from height growth and estimated by using the best linear prediction method (White and Hodge 1989). As a result, 89 of the 550 open-pollinated families, belonging to 45 of the 120 provenances tested, were retained to build the first-generation breeding population. This family selection was then followed by within-family selection for each progeny using a number of phenotypic traits: stem straightness, branch size, branch angle, and tolerance to pests and abiotic stress. At the end, the first-generation breeding population was composed of 360 trees with an average genetic gain close to 20%.

**Figure 1:**
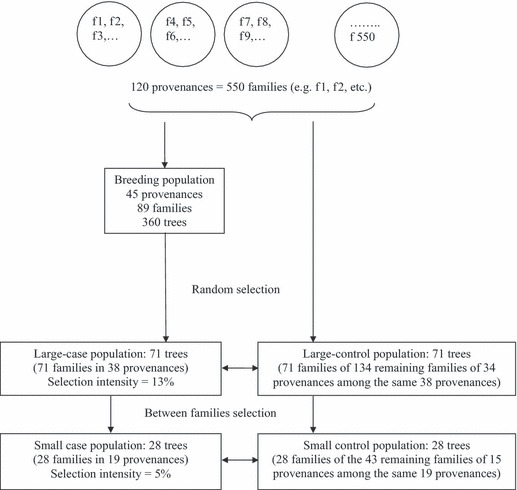
Diagram summarizing the assembly of case and control populations used in this study.

To study the effects of the selection intensity applied to delineate this improved population, a ‘large case population’ was assembled with 71 trees belonging to 38 different provenances (Fig. 1, Table 1) randomly chosen among those making up the first-generation breeding populations described above. This population was composed of the top 13% of the tested open-pollinated families for height growth, representing a genetic gain of 20%. A ‘large control population’ was also assembled with 71 trees belonging to 34 of the 38 natural populations (provenances) from which the first set of 71 trees was assembled (Table 1), but collected in open-pollinated families that had not been subjected to any selection (null selection intensity and genetic gain). To simulate higher selection intensity, a ‘small case population’ was set up with 28 trees chosen from the large case population. These 28 trees belonged to the families with the highest breeding values for height in the large case population; they represented the top 5% of the tested families and had an average genetic gain of 23% over non-improved natural populations. A ‘small control population’ of 28 trees was also assembled from the large control population of 71 trees to control for possible effects related to reduced sampling size. These control trees were chosen from the same natural populations as the 28 selected trees of the small case population, but they had not been subjected to any selection (Fig. 1, Table 1). This population also served to delineate the effect of reducing the sampling size by comparing its genetic diversity patterns with those of the large control population. No comparisons were made between the small case population and either of the two large populations because of the confounding effects of the sampling size and selection intensity. DNA was extracted from the needles of each of the trees using a DNeasy® Plant mini kit according to the manufacturer’s instructions (QIAGEN, Mississauga, Ontario, Canada).

**Table 1:** Geographic location of white spruce provenances and number of trees sampled for each assembled population

					Number of trees per assembled population			
					
Provenance	Province	Latitude North	Longitude West	Altitude(m)	Large control	Large case	Small control	Small case
Beachburg	Ontario	45°42′	76°50′	170	2	1	–	–
Beauceville	Quebec	46°08′	70°49′	213	2	3	2	2
Bois Franc Pierriche	Quebec	46°33′	71°31′	152	2	1	–	–
Canton Blais	Quebec	48°37′	67°17′	167	3	1	–	–
Canton Booth	Quebec	46°47′	78°42′	360	2	1	–	–
Canton Boyer	Quebec	46°35′	75°10′	243	2	5	2	5
Canton Chaumonot	Quebec	47°55′	72°55′	274	2	3	2	1
Canton Cimon	Quebec	48°17′	71°00′	198	3	1	3	1
Canton Dasserat	Quebec	48°13′	79°29′	290	2	1	2	1
Canton Derby	Ontario	44°45′	78°56′	274	2	3	–	–
Canton Desaulniers	Quebec	46°45′	73°05′	365	2	5	2	2
Canton French	Ontario	46°27′	79°10′	304	2	1	–	–
Canton Garin	Quebec	48°22′	65°24′	243	2	1	–	–
Canton Hébécourt	Quebec	48°32′	79°18′	274	2	1	2	1
Canton Laterrière	Quebec	48°05′	71°09′	594	2	2	–	–
Canton Lesage	Quebec	46°20′	75°10′	259	2	2	–	–
Canton McGill	Quebec	46°15′	75°35′	304	2	1	2	1
Carleton	Quebec	48°07′	66°07′	60	2	1	–	–
Cobalt	Ontario	47°20′	79°41′	304	2	1	–	–
Davis Mills	Ontario	45°45′	77°15′	152	1	4	1	2
Estaire	Ontario	46°14′	80°43′	213	2	1	–	–
Foresters Falls	Ontario	45°41′	76°48′	137	–	2	–	–
Havelock	Ontario	44°26′	77°50′	180	1	3	1	2
Irvine Creek	Ontario	45°00′	77°17′	300	3	1	–	–
Kamouraska	Quebec	47°29′	69°58′	30	3	1	2	1
Lac à l’Ours	Quebec	48°46′	71°18′	335	2	2	–	1
Lambton	Quebec	45°56′	71°07′	304	–	1	–	1
Parc Chibougamau	Quebec	48°50′	72°50′	240	–	2	–	1
Parc des Laurentides	Quebec	47°12′	71°14′	730	2	1	2	1
Racine	Quebec	45°30′	72°16′	243	2	2	–	–
Rainy River	Ontario	48°44′	94°32′	323	2	1	–	–
Rutherglen	Ontario	46°17′	79°01′	228	3	1	–	–
Shannonville	Ontario	44°14′	77°15′	90	–	2	–	1
St-Damien-de-Brandon	Quebec	46°20′	73°26′	182	2	2	–	–
Ste-Émilie-de-l’Énergie	Quebec	46°22′	73°43′	396	2	1	–	–
St-Roch-de-Mékinac	Quebec	46°45′	72°46′	152	1	4	1	2
Valcartier	Quebec	46°57′	71°30′	150	2	3	2	1
Whitney	Ontario	45°32′	78°16′	396	3	2	2	1
Total					71	71	28	28

### SNP identification

**Figure 2:**
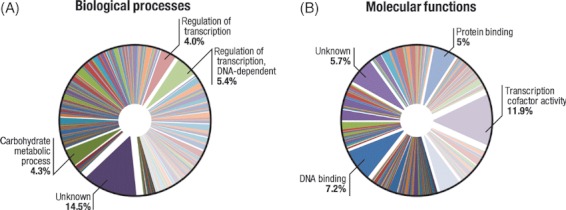
Distribution of the 709 genes carrying single-nucleotide polymorphisms (SNPs) used in this study according to (A) their biological processes and (B) their molecular functions based on their gene ontology (GO) classification.

A total of 1506 SNPs and 30 indels (1–30 bp, from untranslated regions) were chosen for the construction of a large SNP genotyping array. They were located on 822 different genes distributed over the 12 linkage groups of white spruce. This array, called PgLM1, was also used for white spruce gene mapping under the Arborea project (Pelgas et al. 2011). These chosen genes were expressed in different tissues (Pavy et al. 2005) and were representative of a large array of biological processes linked to vital functions such as growth, metabolism, response to stress, defense against pathogens, transcription, and photosynthesis (Fig. 2A). They also represented a large array of molecular functions such as DNA binding, protein binding, hydrolase activity, and transcription cofactor activity (Fig. 2B). Primers for gene amplification and resequencing relied on an assembly of 16 500 unigenes derived from a first-generation white spruce database of about 50 000 expressed sequence tags (ESTs) involved in wood formation, plant growth, and phenology (Pavy et al. 2005). For each gene, coding regions were identified based on alignments with similar sequences from UniProt/SwissProt protein databases. Methods for primer design, PCR, SNP resequencing, and discovery generally followed those of Pavy et al. (2008). For 1416 SNPs and the 30 indels, the polymorphisms were discovered by resequencing the 822 genes from a DNA pool of 24 trees to identify common SNPs (ƒ > 5%; Pelgas et al. 2004). It was also done by sequencing individual white spruce haploid megagametophyte DNA to identify and discard paralogous SNPs showing double peaks in haploid DNA sequence reads (Pelgas et al. 2005, 2006; Pavy et al. 2008). Data management was performed using TreeSNPs (Clément et al. 2010). An additional set of 90 SNPs were also identified *in silico* from the redundancy of EST sequences in white spruce gene clusters following the methods outlined in the study of Pavy et al. (2006).

### SNP genotyping

Genotyping of the 142 sampled individuals was performed by constructing a 1536-SNP bead array (PgLM1) and using the Illumina GoldenGate SNP genotyping assay (Illumina, San Francisco, CA, USA; Fan et al. 2003; Shen et al. 2005). This array had been previously used to map genes (Pelgas et al. 2011). The GoldenGate assay consists in genotyping genomic DNA by hybridizing two allele-specific (ASO) and one locus-specific (LSO) oligos with each DNA sample in the array matrix. The 1506 SNPs and 30 indels were genotyped in 96-well plates using 2 μg of DNA extract normalized at 50 ng/μL for each sample. Genotyping was conducted at the Genome Quebec Innovation Centre (team of A. Montpetit, McGill University, Montreal, Canada). The GenTrain score was used to evaluate the accuracy and efficiency of SNP genotyping. This score reflects the degree of separation between homozygote and heterozygote clusters for each SNP (Fan et al. 2003). The lowest acceptable score was set at 0.25, similar to the conservative criterion used in human genetic studies (http://www.illumina.com; Fan et al. 2003) and in previous genome scan studies relying on this assay in white spruce (Namroud et al. 2008; Pavy et al. 2008; Beaulieu et al. 2011; Pelgas et al. 2011). Further details on the assay can be found in Fan et al. (2003) and Shen et al. (2005). DNA reports, locus summaries, and the data set were generated using the genotyping module of the BeadStudio data analysis software (Illumina). The repeatability of the genotyping assay was evaluated using 14 positive controls.

### Data analysis

To determine the extent to which selection intensity affected genetic diversity, we compared a number of genetic diversity estimates between case and control populations of small size and between case and control populations of large size. To further determine the effect of sampling size, the genetic diversity estimates were compared between the small and large control populations. These genetic diversity estimates included the percentage of polymorphic SNPs (*P*_O_) at the 95% level, the average number of alleles per locus (*A*), observed heterozygosity (*H*_O_), expected heterozygosity or gene diversity (*H*_E_) corrected for small samples according to Nei (1978), the deviation of genotype frequencies from Hardy–Weinberg equilibrium estimated by the within-population fixation index (*F*_IS_), and allele frequencies for each SNP. Moreover, alleles were grouped into 10 classes based on their frequencies in each population, which made it possible to compare the distribution of allele frequency classes between populations. Alleles with frequencies lower than 5% were defined as rare. The heterogeneity of *H*_O_*, H*_E_, and *F*_IS_ between populations was tested with paired *t*-tests using the statistics package of the software R version 2.6.1 (http://www.r-project.org). A Fisher’s exact test and a chi-square test (*χ*^2^-test) were used to check the heterogeneity of allele frequencies for each SNP and the distribution of allele frequency classes between populations, respectively. The same parameters were used to assess the effect of increasing the selection intensity by comparing same-size control and case populations: small case versus small control and large case versus large control populations.

Among-population genetic differentiation between the population pairs in each comparison mentioned above was estimated using the parameter (*θ*_RH_). This parameter was proposed as an estimator of *F*_ST_ by Robertson and Hill (1984) and was modified to account for low to moderate population differentiation by Raufaste and Bonhomme (2000). The significance of *F*_IS_ and *F*_ST_ (we use the term *F*_ST_ to indicate *θ*_RH_) was tested with 10 000 permutations of alleles within populations and of samples between populations, respectively. All the genetic parameters were obtained and statistical tests conducted using Genetix version 4.05 (http://www.genetix.univ-montp2.fr/genetix/genetix.htm; Belkhir et al. 1996–2004), except for the Fisher’s exact tests and chi-square tests that were performed with SAS 9.0 (SAS Institute Inc., Cary, NC, USA).

## Results

### Genotyping success

Among the 1506 SNPs and 30 indels submitted to multiplex genotyping, 1234 SNPs and 21 indels (from 1 to 6 bp) were successfully genotyped with a GenTrain score higher than the conservative threshold of 0.25 set for this study (Table 2) and with <1% missing data per SNP scored, on average (average call rate of 99.5% with lowest call rate at 95% for any given SNP). Based on the positive controls, the repeatability of the genotyping assay was estimated at 99.95%. The 272 SNPs and nine indels that failed to reach the threshold were considered non-reliable and simply discarded from further analysis. Another 121 SNPs that were monomorphic among all samples were also discarded from analysis because we could not ascertain whether their monomorphism was attributable to the failure of one of the ASOs in the GoldenGate assay or to the fixation of the corresponding alleles in the populations, in which case they would not be useful for the comparative analysis of genetic diversity. This left us with a total of 1113 valid SNPs and 21 valid indels for comparative analysis (total of 1134 polymorphisms), which represented 74% of the markers originally submitted to the genotyping assay (Table 2). This success rate for newly genotyped markers was marginally higher than the ones obtained previously (67.0% and 66.5%) using the same GoldenGate assay and two different SNP arrays (PgLM1, Namroud et al. 2008 and Pavy et al. 2008; PgWD1, Beaulieu et al. 2011). The present group of markers represented 709 genes distributed over the 12 linkage groups of the white spruce genome (Pelgas et al. 2011) or 86% of the original set of SNP-bearing genes submitted to the genotyping assay. The present success rate was marginally higher than that obtained when genotyping a pedigree gene mapping population with the same SNP array (Pelgas et al. 2011), given that 34 of the present valid SNPs did not segregate in the mapping population.

**Table 2:** Genotyping success of gene single-nucleotide polymorphisms (SNPs) using the Illumina GoldenGate multiplex assay

Gentrain score*	Total number of SNPs assayed	Number of SNPs showing no polymorphism	Number of segregating SNPs considered for analysis
≤0.25 (failed)	281	–	–
0.25–0.30	18	1	17
0.30–0.40	68	23	45
0.40–0.50	95	23	72
0.50–0.60	169	46	123
0.60–0.70	201	13	188
0.70–0.80	484	15	469
0.80–0.90	219	0	219
0.90–1.00	1	0	1
Total	1536†	121	1134‡

* According to the study of Fan et al. (2003).

† Including 30 indels of 1–30 bp.

‡ Representative of 709 genes and including 21 indels of 1–6 bp.

### Sampling effects

All parameters (*P*_O_, *A*, *H*_O_ and *H*_E_) were comparable between the two control populations of different sizes (Table 3), including *F*_IS_, which was lower in the small control population compared with the large one but not significantly different (*P >* 0.05), as tested using 1000 bootstraps over loci. A total of 14 alleles were lost in the small control population as compared with the large control population (Table 3). However, these lost alleles had a low minor allele frequency (average MAF of 0.017) and represented less than one percent of the alleles present in the large control population. The overall distribution of allele frequency classes did not significantly vary between the two control populations (*χ*^2^ = 3.5; *P* = 0.94), including rare alleles with MAF ≤ 0.05 (*χ*^2^ = 0.4; *P* = 0.52). When considering each SNP individually, none showed significant differences in allele frequencies between the two control populations after correction for multiple testing using the false discovery rate (FDR) (Storey and Tibshirani 2003), at a relaxed confidence level of *Q* ≤ 0.10. Before correction, five SNPs showed significant differences at *P* ≤ 0.05, but none remained significant at *P* ≤ 0.01. Similarly, the genetic differentiation (*F*_ST_ = −0.0061) between the two populations was not significantly different from zero.

**Table 3:** Genetic parameters of the four experimental populations of white spruce*

Population	Number of trees	Average breeding value (m)†	Number of polymorphic SNPs	*P*_O_	*A*	Number of rare alleles *(f* < 0.05)	*H*_O_	*H*_E_ (unbiased)	*F*_IS_
Large control	71	0.03 ± 0.21‡	1134	0.82	1.99	331	0.282 ± 0.169	0.282 ± 0.160	−0.0007 ± 0.0055§
Large case	71	0.47 ± 0.21	1102	0.83	1.98	316	0.280 ± 0.168	0.280 ± 0.158	−0.0012 ± 0.0055
Small control	28	0.06 ± 0.22	1134	0.83	1.94	317	0.284 ± 0.183	0.280 ± 0.166	−0.0143 ± 0.0078¶
Small case	28	0.56 ± 0.21	1102	0.83	1.94	316	0.283 ± 0.181	0.278 ± 0.164	−0.0161 ± 0.0071¶

* *P*_O_: percentage of polymorphic loci (95% level); *A*: average number of alleles per single-nucleotide polymorphisms (SNP); *H*_O_: average observed heterozygosity; *H*_E_: average unbiased expected heterozygosity (Nei 1978); *F*_IS_: average within-population inbreeding coefficient.

† The average breeding value is the difference between the average height of the families included in each population and that of all the tested families expressed in meters and measured at 15 years.

‡ Standard deviation.

§ Standard deviation estimated using 1000 bootstraps based on SNPs.

¶ Significant, *P* ≤ 0.05 using 10 000 permutations.

### Effects of selection intensity

**Figure 3:**
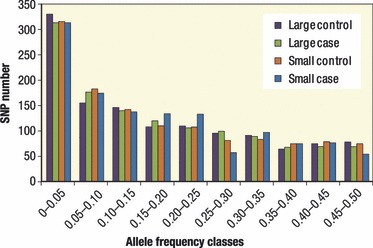
Single-nucleotide polymorphisms (SNP) distribution among 10 allele frequency classes for the two case and two control populations of white spruce.

All genetic diversity estimates (*P*_O_, *A, H*_O_, *H*_E_ and *F*_IS_) were similar between same-size case and control populations and did not show any significant statistical difference (*t-*tests; *P* > 0.05) (Table 3). Also, the overall distribution of allele frequency classes was not significantly different between the same-size populations compared (*χ*^2^ = 3.5 and 14.3; *P* = 0.84 and 0.94, respectively, for the large- and small-size populations), including the proportion of rare alleles with MAF ≤ 0.05 (Table 3; Fig. 3; *χ*^2^ = 0.4 and 0; *P* = 0.52 and 0.29, respectively). When tested for each SNP individually, allele frequencies were not significantly different after correction with FDR even when we relaxed the confidence level to *Q* ≤ 0.10. Before correction, 36 and 38 SNPs were significantly different at *P* ≤ 0.05 between the two small (*n* = 28) and between the two large populations (*n* = 71), respectively. This sizeable number of significant SNPs before correction for multiple testing certainly contains false positives, but nine and ten SNPs maintained significant differences at a higher probability (*P* ≤ 0.01; Table 4) between the two small and between the two large populations, respectively, thus reflecting possible effects from applying selection. Genetic differentiation (*F*_ST_) was ten times higher between the two small than between the two large populations (0.0022 and 0.0002, respectively). While the differentiation between the two large populations was not significantly different from zero, that between the two small populations was significantly greater than zero, as tested using 1000 bootstraps over SNPs.

**Table 4:** List and properties of single-nucleotide polymorphisms (SNPs) potentially affected by artificial selection identified with Fisher’s exact test at a minimum confidence level of *P* ≤ 0.01*

SNP	Populations compared	Locus†	*e*-value‡	Gene family	Putative biological function§	Position	*P*-value (Fisher’s exact test)	*Q*-value (false discovery rate)	*F*_ST_¶	*P-*value (*F*_ST_)	*P-*value/PVE** (association with breeding value)
*08Pg03931f*	Large case vs Large control	*AT5G54160*	1.00E-63	O-Methyltransferase 1	Lignin composition	Exon (synonymous)	0.001	1.000	−0.004	1.0	0.0399/4.5
*pa08Pg12255f*††	Large case vs Large control	*AT3G13750*	4.00E-102	Beta galactosidase 1 (BGAL1)	Cell elongation, fruit ripening and storage mobilization, functional stability of the wall during cell death as cotyledons undergo senescence	Intron (N/A)	0.005	1.000	−0.004	1.0	0.0174/4.6
*i08pg01084a*††	Large case vs Large control	*AT3G22960*	0	Pyruvate kinase	Glycolysis regulation	Exon (N/A)	0.006	1.000	0.667	1.0	0.0804/3.6
*10614t2*††	Large case vs Large control	N/A	N/A	N/A	N/A	Exon (non-synonymous)	0.006	1.000	0.038	0.015	0.3944/1.3
*08pg02707e*	Large case vs Large control	*AT5G48900*	2.00E-106	Pectate lyase family protein	Fruit ripening, cell wall degradation	3′ UTR	0.007	1.000	0.000	1.000	0.0012/9.2
*08pg10691j*	Large case vs Large control	*AT3G07720*	3.00E-113	Kelch repeat-containing protein	Role in oogenesis, organization of cytoskeletal, plasma membrane or organelle structures, coordination of morphology and growth in yeast cells	Exon (synonymous)	0.008	1.000	0.003	0.349	0.1324/2.9
*08pg02707f*	Large case vs Large control	*AT5G48900*	2.00E-106	Pectate lyase family protein	Fruit ripening, cell wall degradation	3′ UTR	0.008	1.000	0.000	1.0	0.0263/5.1
*Py08pg10659-2*††	Large case vs Large control	*AT3G12780*	0	Phosphoglycerate kinase (PGK1)	Glycolysis	Exon (non-synonymous)	0.008	1.000	0.002	0.384	0.0979/3.3
*08pg04210d*	Large case vs Large control	*AT1G48110*	1.00E-92	Evolutionarily conserved C-terminal region 7(ECT7)	Plant development	Exon (non-synonymous)	0.009	1.000	−0.001	0.489	0.0493/2.7
*08Pg00936e*	Large case vs Large control	*AT5G65700*	7.00E-77	Barely any meristem 1 (BAM1)	Leaf development, male gametophyte development, ovule specification, and function	Exon (non-synonymous)	0.009	1.000	−0.007	1.0	0.3981/1.5
*08pg02761g*	Small case vs Small control	*AT4G24780*	7.00E-123	Pectate lyase family protein	Fruit ripening, cell wall degradation	Exon (synonymous)	0.001	0.648	0.034	0.124	0.0009/26.7
*09121m*††	Small case vs Small control	*AT5G20510*	7.00E-99	ALFIN-like 5 (AL5)	Abiotic stress and development	3′ UTR	0.001	0.648	0.043	0.097	0.0032/19.5
*08pgsb29b*††	Small case vs Small control	*AT1G77450*	3.00E-18	Arabidopsis NAC domain containing protein 32	Embryonic, floral, and vegetative development, lateral root formation and auxin signalling, defense and abiotic stress.	Exon (synonymous)	0.005	0.971	0.132	0.005	0.1149/7.8
*08Pg05193f*	Small case vs Small control	*AT2G38120*	2.00E-165	Auxin-resistant 1 (AUX1)	Root development, response to nematode	Intron (N/A)	0.006	0.971	0.129	0.005	0.0008/23.6
*08Pg05193h*	Small case vs Small control	*AT2G38120*	2.00E-165	Auxin-resistant 1 (AUX1)	Root development, response to nematode	Intron (N/A)	0.006	0.971	0.129	0.004	0.0008/23.6
*Py08pg10192*	Small case vs Small control	*AT2G32910*	2.00E-41	N/A	N/A	3′ UTR	0.007	0.971	0.123	0.011	0.0680/9.7
*08pgsb08a*	Small case vs Small control	*AT5G38650*	6.00E-49	Proteasome maturation factor UMP1 family protein	Maturation of the 20S proteasome	Intron (N/A)	0.007	0.971	0.123	0.004	0.0338/12.0
*08pgsb08b*††	Small case vs Small control	*AT5G38650*	6.00E-49	Proteasome maturation factor UMP1 family protein	Maturation of the 20S proteasome	Exon (synonymous)	0.007	0.971	0.123	0.002	0.0338/12.0
*Py08pg9772-1*	Small case vs Small control	*AT1G08510*	3.00E-112	Fatty acyl-ACP thioesterases B (FATB)	Plant growth and seed development	3′ UTR	0.008	0.971	0.120	0.010	0.1624/6.6

* SNPs with *P* > 0.01 were not included because they are more likely to be false positives, as explained in the Discussion.

† SNP annotations and nomenclature are as in the study of Pavy et al. (2008).

‡ Related transcript sequences can be found upon request in the Arborea database at http://www.arborea.ulaval.ca.

§ Biological functions were based on literature research for the GO annotations obtained for each locus through BLASTx searches against *Arabidopsis* blast sets database at http://www.arabidopsis.org/tools/bulk/go/index.jsp.

¶ *F*_ST_ between case and control populations was calculated as an estimate of *θ*_RH_ proposed by Robertson and Hill (1984) and corrected by Raufaste and Bonhomme (2000).

** *P-*value of association tests between the SNP and the breeding value for height at 15 years of age, and percentage of variation explained (PVE). The tests were carried out using the GLM procedure available in the TASSEL software (http://www.maizegenetics.net/tassel) and in combining both the control and the case populations for each set.

†† SNP associated with a quantitative trait loci (QTL) for height growth and/or bud phenology in white spruce (see Pelgas et al. 2011).

## Discussion

### Impact of reducing population size

The decrease in population size between large and small control populations (about 60% in the small control compared with the large control population) induced a slight increase in the percentage of polymorphic loci and, at the same time, a slight decrease in the average number of alleles per locus and the total number of rare alleles in the small control population. However, all these changes were not statistically significant and not sufficient to induce a significant change in the frequencies of any SNP or in the overall distribution of allele frequency classes, even for rare alleles (MAF ≤ 0.05). Thus, genetic diversity was not reduced in the small control population, relative to the large control one. These findings could possibly be explained in two interconnected ways. The first explanation can be linked to the way the small control population was set up. In the present study, the individuals in the small control population were not selected randomly among the provenances of the large control population, as expected under genetic drift. They were rather selected within provenances that also included families with the highest breeding values for height, because the objective was to set up a control population for the small case population with the same geographic background, so as to neutralize the possible confounding effect of different geographic backgrounds between small case and large case populations. Consequently, the sampling scheme used should not be considered as strictly equivalent to a simulation of genetic drift. One could also argue that, because the size of both control populations was much lower than the effective size of natural populations they originated from, a bottleneck effect might be already associated with the large control population. To address this potential issue, we compared the level of genetic diversity of the large control population (size of 71) with that of a set of 158 trees representative of natural populations in the same geographic area, assembled by Jaramillo-Correa et al. (2001) and further genotyped by Namroud et al. (2008) for 534 SNPs of 345 genes. Overall, 197 SNPs were in common between the study of Namroud et al. (2008) and the present study. The genetic diversity parameters were similar (*H*_O_ = 0.354 and 0.344, and *H*_E_ = 0.347 and 0.343, for the populations of 158 and 71 trees, respectively), the difference being well within the standard errors of estimates. Only one of the alleles was lost in the large control population (71) as compared with the population of Namroud et al. (2008), and the frequency of that allele in the latter population was below 1%, thus a rare variant. Therefore, even if this comparison does not correct for the fact that the large control population was not randomly chosen, the genetic diversity that it contains was likely representative of the species natural populations.

The second explanation comes from the fact that families having the highest breeding values belong to natural populations that also had higher heterozygosity on average. We must recall that to make the small case and control populations comparable in terms of background origins, control trees were drawn from the same populations as those of selected trees. Thus, increasing the selection intensity to assemble the small case population, an indirect selection was made for a population with higher heterozygosity, thus resulting in increased heterozygosity for the small control population as well. This argument is illustrated by the average expected heterozygosity of 0.245 (SE = 0.023) for the 43 trees of the large control population that were not selected to be part of the small control population, whereas that of the 28 trees making up the small control population was 0.319 (SE = 0.028). As a consequence, a significant excess of heterozygotes (*F*_IS_) compared with what was expected from Hardy–Weinberg equilibrium was also observed in the small control population (Table 3).

### Impact of increasing the selection intensity

The comparison of same-size case and control populations did not result in notable differences in standard genetic diversity estimates, namely the proportion of polymorphic loci (*P*_O_), the number of alleles per locus (*A*), heterozygosity (*H*_O_ and *H*_E_), *F*_IS_, and the frequency of rare alleles. It is likely that biallelic markers such as those used in the present study do not offer much sensitivity for identifying differences in some of these genetic diversity parameters on a per locus basis, although a large number of loci was sampled, which should have led to high power in detecting differences in heterozygosity. However, we noticed that when retaining a smaller proportion of families under the tested scenario of high selection intensity (down to the best 5%), a small but proportionally large tenfold increase in genetic differentiation (*F*_ST_) was induced between the small case and control populations, compared with the scenario where the best 13% of the families were retained (large case versus control populations). This increase in overall genetic differentiation is significant and indicates that even in species highly diversified genetically such as spruces and even at an early stage of domestication, a sample of much reduced size resulting from high selection intensity can lead to increased genetic differentiation.

Selection can be effective in altering gene frequencies if there is a strong correlation between the phenotype and the genotype, and more so if the character is affected by a small number of genes (Falconer and MacKay 1996). However, most of commercial traits of interest are thought to be affected by a large number of genes (Lynch and Walsh 1998), and there is evidence that tree height and related traits such as bud flush and budset in white spruce are controlled by a large number of genes located on several linkage groups, each one having small genetic effects (Pelgas et al. 2011). In a recent study, these authors reported 52 distinct quantitative trait loci (QTLs) linked to height growth, each explaining between 2.5% and 10% of the variation observed in that quantitative trait, while 85 QTLs were related to phenological traits. A similar pattern is also emerging for gene polymorphisms related to wood characters in white spruce association genetic studies, with percent of phenotypic variance explained by individual marker loci being usually low (Beaulieu et al. 2011). It is interesting to note that in the present study, even if a high selection intensity was applied to such a trait as growth, which is controlled by numerous genes each with small effects, it did not result in a significant impact on allele frequencies when a correction for multiple testing was applied. However, as shown below, when the statistical threshold is relaxed, an important fraction of the SNPs found to be putatively affected by selection was found on genes linked to the sub-mentioned QTLs related to growth and phenology traits.

Even if the tested selection intensities did not change significantly allele frequencies, it is expected that it may induce an increase in linkage disequilibrium (LD), which will generally reduce the additive genetic variance usable for gains in the future (Mueller and James 1983). Such a reduction will generally predominate (Bulmer 1971) unless epistatic effects are large (Griffing 1960), as gene interactions play a role in causing the additive effects of alleles to change as the genetic composition of the population changes (Barton and Keightley 2002). Moreover, covariance of allelic effects can also arise under non-random gametic association in the progeny if mating is not random in the selected tree population. Given that natural populations of white spruce harbor rapid decay of LD at very short distances well within gene limits (Namroud et al. 2010; Beaulieu et al. 2011; Pavy et al. 2012), such an effect, if real, should be discernable in the white spruce genes surveyed.

To test whether LD might have been induced in the selected populations, we estimated unphased LD between each pair of SNPs within genes, i.e., for genes for which more than one SNP had been mined, using the squared allelic correlation coefficient (*r*^2^) as a measure of LD. It was possible to estimate *r*^2^-values for 205 pairs of SNPs. The average *r*^2^ values were similar for each of the four populations and not significantly different (*F* = 1.31, *P* = 0.27), ranging from 0.319 to 0.333. For three of the SNPs potentially affected by artificial selection in the large populations (Table 4) and for which we had information on LD, a slight increase in LD was noted for one of them, *08pg10691j*, where *r*^2^ = 0.0787 for the control population increased to *r*^2^ = 0.1336 in the case population. However, for the two other SNPs *08pg02707e* and *08Pg00936e*, the LD was slightly reduced in the large case population as compared with the control population (from *r*^2^ = 0.4961 to *r*^2^ = 0.4454 for *08pg02707e*, and from *r*^2^ = 0.0084 to *r*^2^ = 0.0061 for *08Pg00936e*). For the set of small populations, LD decreased for the two SNPs potentially affected by selection for which we had *r*^2^ estimates: for *08pg02761g*, *r*^2^ decreased from 0.3749 to 0.1532 for control and case populations, and for *09121m*, *r*^2^ respectively, decreased from 0.3819 to 0.2396. Thus, evidence for any increase in LD after artificial selection was very weak in the present study. However, the present study design was not optimal to obtain accurate estimates of LD. Only a large series of candidate genes potentially affected by selection with a good coverage of SNPs evenly distributed along the gene sequences would make it possible to obtain sound estimates of LD and test the hypothesis of increase in LD after selection. At the same time, larger population sizes would be required while maintaining selection intensities to increase statistical power in detecting significant shifts in LD.

### Heterozygosity excess

An important trend specific to the small case population analyzed was the significant excess of heterozygotes (*F*_IS_) it harbored compared with expectations from Hardy–Weinberg equilibrium. At a first glance, this excess combined with the increase in genetic differentiation between the small case and the small control populations may support the hypothesis of a positive relationship between heterozygosity and growth, survival or fitness. This trend has been observed for knobcone pine (Strauss 1986), Chir pine (Sharma et al. 2007), and Norway spruce (Bergmann and Ruetz 1991). Others have suggested that a possible underlying overdominance of the loci responsible for species fitness could be at the origin of the positive correlation between heterozygosity and species fitness and related traits (Mitton and Grant 1984; Smouse 1986; Zouros and Foltz 1987). To test this hypothesis, we used the GENHET R-function (Coulon 2010) to estimate two parameters for individual heterozygosity: the internal relatedness IR (Amos et al. 2001) and the homozygosity by locus HL (Aparicio et al. 2006). No significant correlation (Kendall’s τ > 0.05) could be observed between these parameters and growth expressed by the height of 15-year-old trees in the large or small case population. Consequently, the extent to which this factor can account for the conservation of these alleles and higher heterozygosity remains unknown, especially considering that other studies provided inconclusive results (e.g., Ledig et al. 1983; Savolainen and Hedrick 1995; Deng and Fu 1998). Moreover, the size of our small case and control populations might have been too limited to provide strong evidence of the presence of such a relationship, considering that it is difficult to detect in the absence of inbreeding (Hansson and Westerberg 2002).

### Potential candidate SNPs and putative roles

The statistically non-significant differences between SNP allele frequencies (even after relaxing the FDR criterion to *Q* ≤ 0.10) in all population comparisons after correction for multiple analyses were not surprising. The FDR method provides an increased power to detect differentiation between paired samples but remains conservative, although less conservative than the Bonferroni correction (Narum 2006). At the same time, the large number of significantly different SNPs observed before correction at *P* ≤ 0.05 between the two large populations (38), and the two small populations (36), cannot be explained biologically and may contain a fair proportion of false positives. Discarding all these SNPs would nonetheless likely eliminate valuable information about some true-positive SNPs. Therefore, we increased the threshold level to *P* ≤ 0.01, which allowed us to reduce the possible number of false-positive SNPs while resulting in the identification of a small number of significantly differentiated SNPs that could be potentially affected by selection.

Previous studies that used standard differentiation tests were based on enzyme markers (allozymes) and detected only one or a few significantly different loci (e.g., Knowles 1985; Cheliak et al. 1988; Desponts et al. 1993; Rajora 1999) or one or few lost alleles (e.g., Chaisurisri and El-Kassaby 1994; El-Kassaby and Ritland 1996) between the breeding (selected) and natural populations. These figures are comparable to those obtained after increasing the confidence level to *P* ≤ 0.01 in our study, where 9 and 10 SNPs remained significant in the pairwise comparisons between case and control populations of the same size, although the number of SNPs and loci tested was much larger. These numbers were equivalent to a proportion of about 1% of the total number of SNPs analyzed, which was lower than that obtained when detecting SNPs under natural selection with outlier-detection analyses relying on summary-statistics methods in wild populations. For instance, Namroud et al. (2008) reported 3.7% of the SNPs to be potential candidates for selection among natural populations of white spruce using a summary-statistic outlier-detection method based on population differentiation. Prunier et al. (2011) reported 4.5% of the SNPs as outliers among climatic groups in black spruce using a similar approach. Even if artificial selection coefficients were larger in the present study by an order of magnitude than that estimated for outlier SNPs in natural spruce populations (Prunier et al. 2011), this trend should not be surprising, given that natural populations have been subjected to selection for a large number of generations since their post-glacial establishment during the Holocene many thousands of years ago (Prunier et al. 2011). In the present study, only one generation of artificial selection could be tested.

It was interesting to observe that all genes carrying SNPs that remained significant at *P* ≤ 0.01 could be related to growth and reproductive processes and, to a lesser extent, to plant response to biotic or abiotic stress (Table 4). In particular, five SNPs that remained significantly different between the small case and the small control populations were involved in a number of vital biological functions. For example, one SNP (*08Pg02761*) significantly different between the small case and the small control population belongs to a gene of the pectate lyase family, which is often linked to plant growth, development, and response to chemical stimuli (Taniguchi et al. 1995; Wu et al. 1996). Two other SNPs (*08pgsb08a* and *08pgsb08b*) belong to a gene from the proteasome maturation factor UMP1 family protein, which is involved in the proteasomal degradation pathway. It is essential for many cellular processes, including the cell cycle, the regulation of gene expression, and responses to oxidative stress (Aiken et al. 2011). This gene was found to be associated with a QTL involved in height growth in white spruce (Table 4, and additional files 4, 5 and 6 in Pelgas et al. 2011). Six other significant SNPs also belonged to genes found to be significantly linked to genomic regions underlying growth and phenology traits (Table 4), and one of them (*10614t2*), which pertains to an arabino-galactan protein gene reported to be involved in wood formation in *Pinus taeda* L. (Loopstra and Sederoff 1995), has also been reported to be involved in local adaptation in a genome scan aiming to detect gene SNPs significantly differentiated among white spruce natural populations (Namroud et al. 2008).

Among the seven SNPs found significant in the present study and whose corresponding genes were also reported to be associated with QTLs (Pelgas et al. 2011), four were detected by comparing case and control larger populations, while the three remaining SNPs were found by comparing case and control smaller populations. The QTLs associated with the three latter SNPs are located on three different linkage groups, i.e., 4, 10, and 11 (Pelgas et al. (2011). Among the first group of four SNPs, two are located on linkage group 8 (*i08pg01084a* and *10614t2*) but about 100 cM apart. The two other QTLs are on linkage groups 2 and 5. Hence, these SNPs are well dispersed throughout the genome, and their corresponding QTLs are all distinct.

Results of association tests between the breeding values for height and SNPs potentially affected by selection for both sets of populations (large and small) also strengthen our belief that these SNPs are throughly affected by selection. Indeed, among the 10 detected SNPs in the pair of large control and large case populations, five were significant at *P* ≤ 0.05, and two more at 0.05 > *P* ≤ 0.10. Each of them could explain between 3% and 9% of the total variation in breeding values. Similarly, for the set of the two small populations, seven of the nine detected SNPs were found significantly associated with breeding values for height at *P* ≤ 0.10 and six at *P* ≤ 0.05, and up to 27% of the observed variation in breeding values could be explained by the most significant SNPs (Table 4). While some of these SNPs might have potential predictive value for marker-assisted selection, these and their effects on phenotypes would need to be validated in large association genetics populations, where much smaller percents of variation explained are usually observed (Beaulieu et al. 2011). One additional interesting finding is that the SNPs that are potentially affected by artificial selection in the current study (Table 4) have intermediate allele frequencies (0.10 > *f* < 0.50). This trend provides further support to the idea that they are indeed affected by selection, given that the SNPs expected to be the most influenced by selection have been predicted to be those harboring intermediate frequencies (Namkoong 1979).

The SNPs that were identified in the present study may also be of special interest in future breeding programs for white spruce, and it is possible that they will exhibit a higher level of differentiation in future generations. A more accentuated genetic differentiation (*F*_ST_) in neutral genetic markers has already been observed between the second generation of Douglas-fir orchards and their wild progenitors compared with the corresponding first generation (El-Kassaby and Ritland 1996). With the two selection intensities simulated in the present study, we did not observe a reduction in or loss of genetic diversity. A follow-up in advanced selection generations remains necessary to determine whether these significantly different SNPs will also exhibit significant genetic differentiation in future generations, whether new outliers will be uncovered and whether genetic diversity will be maintained as selection intensity increases.

Comparison with previous studies is not an easy task because in addition to using a limited number of loci (mainly neutral), most studies did not provide clear figures about the selection intensity that their breeding populations experienced. Authors also based their conclusions upon the comparisons of populations of different sizes (e.g., Muona and Hariu 1989; Bergmann and Ruetz 1991), groups of natural and breeding populations with different average population sizes (e.g., El-Kassaby and Ritland 1996), or unbalanced numbers of populations for the natural and breeding populations (e.g., Chaisurisri and El-Kassaby 1994; Rajora 1999). Williams et al. (1995) compared the impact of two breeding strategies, multiple populations versus hierarchical, on loblolly pine genetic diversity using isozymes. They concluded that there was no specific genetic pattern induced in the diversity of loblolly pine when the selection intensity increased from the first to the third generation. However, by comparing samples belonging to different generations but of the same size in their study (21–25 samples, their Table 3), one can easily notice the slight increase that occurred between such samples in terms of heterozygosity between the second and third generations (Table 4 in Williams et al. 1995). Unfortunately, the lack of information accurately documenting the selection intensity in each of these generations while controlling for the population size makes it difficult to directly compare these results with our data. In a simulation study, Danusevicius and Lindgren (2005) suggested that the optimum breeding population size should range between 30 and 70 for northerly coniferous species if we are to simultaneously consider the advance in breeding value, the associated loss of gene diversity, and the time and cost components of long breeding populations. Although some attempts have been made to determine the relationship between breeding population size and genetic diversity (e.g., Maruyama and Fuerst 1985; Danusevicius and Lindgren 2005), empirical studies controlling the size of the breeding populations and documenting the selection intensity are still largely needed to confirm the nature of the occurring changes.

## Conclusion

The main contribution of this study consisted in surveying a much expanded sample of the expressed plant genome in assessing the effects of artificial selection on natural genetic diversity. While no significant loss in genetic diversity was noted after selecting for one generation, subtle effects were nevertheless observed, implicating differentiation of allele frequencies at certain gene loci and significant associations with phenotypic selection criteria. Whether these SNPs may harbor good predictive value of breeding values in marker-aided selection schemes remains to be verified in large association populations. As for the issue of gene conservation, previous studies suggested that 30 to 70 individuals should be sufficient to ensure that the genes most influenced by selection (i.e., allele frequencies in the intermediate range, Namkoong 1979) and of primary importance for genetic gain in the first five to ten generations would be maintained in the breeding population (Johnson et al. 2001). In the present study, we have shown that an artificially selected white spruce population as small as 28 trees corresponding to a proportion of selected families of 5% essentially maintained the genetic diversity found in the control population. Such a population size does not make it possible to maintain all very low-frequency alleles that might be important over the long term, as we noticed from the loss of such 14 rare frequency alleles from sampling. However, this issue can be addressed by conserving independently gene resource populations. Determining the threshold at which genetic diversity levels will be significantly reduced presents an interesting approach that should allow breeders to make informed decisions regarding the management of breeding populations as well as gene resource populations.

## Data archiving statement

Data are deposited in the Dryad repository: doi:10.5061/dryad.np8708k2.
